# An experience of mass administration of fractional dose inactivated polio vaccine through intradermal needle-free injectors in Karachi, Sindh, Pakistan

**DOI:** 10.1186/s12889-020-10041-8

**Published:** 2021-01-06

**Authors:** Umar Farooq Bullo, Jaishri Mehraj, Syed Musa Raza, Shumaila Rasool, Noreen Naz Ansari, Ahmed Ali Shaikh, Zamir Ali Phul, Sohail Ahmed Memon, Rehan Iqbal Baloch, Zahoor Ahmed Baloch, Shoukat Ali Chandio

**Affiliations:** 1 Emergency Operations Centre for Polio Eradication and Immunization, Government of Sindh, Karachi, Sindh Pakistan; 2 Shaheed Zulfikar Ali Bhutto Institute of Science and Technology (SZABIST), Karachi, Sindh Pakistan; 3 The United Nations Children’s Fund (UNICEF), Karachi, Sindh Pakistan; 4 National Stop Transmission of Polio (N-STOP) Program, Karachi, Sindh Pakistan; 5 Bill and Melinda Gates Foundation, Karachi, Sindh Pakistan; 6 Expanded Program on Immunization, Government of Sindh, Karachi, Sindh Pakistan

**Keywords:** Inactivated polio vaccine, Fractional dose, Vaccination, Campaign, Pakistan

## Abstract

**Background:**

Inactivated Polio Vaccine (IPV) campaign was conducted in February 2019 in Karachi where needle-free injectors were introduced for the administration of the fractional dose of IPV (fIPV) on a large scale. This study aimed to determine the impact of needle-free injectors on vaccination coverage.

**Methods:**

In four towns of Karachi, fIPV was given using needle-free injectors “PharmaJet Tropis ID”. Whereas, in six towns full dose of IPV was administered to children of 4–59 months of age. Cluster surveys through rapid convenience assessment method were conducted after the completion of vaccination activity.

**Results:**

A total of 33,815 households’ data was analyzed. Among these, 27,650 (82.8%) children were vaccinated. In fIPV areas, 85.3% of children were vaccinated compared to 79.5% in full dose IPV areas. A comparison of reasons for unvaccinated showed that 1.6% of parents do not give importance to vaccination in fIPV areas compared to 4.2% in full IPV areas (*p*-value < 0.0001). More children were not vaccinated due to fear of injection 1.8% in full IPV areas compared to 0.7% in fIPV areas (*p*-value < 0.0001). The source of campaign information shows that more frequent mobile miking 3.1% was observed in fIPV areas compared to 0.4% in full IPV areas (*p*-value < 0.0001).

**Conclusions:**

Our analysis supports the fractional dose of IPV in mass campaigns to achieve good vaccination coverage especially using needle-free injectors “PharmaJet Tropis ID” and vigorous social mobilization activities are expedient in accomplishing high coverage.

## Background

Polio cases are still reported from two endemic countries; Afghanistan and Pakistan [1]. Uninterrupted virus circulation remains a constant public health challenge for the state. Rigorous efforts have been made since the declaration of the polio program as a national emergency. The use of Inactivated Polio Vaccine (IPV) is recommended in the campaign during endgame strategies to increase population immunity in advance of withdrawal of Oral Polio Vaccine (OPV) serotype, eradicate remaining wild-type polioviruses, and respond to any outbreaks of vaccine-derived poliovirus [2, 3]. Since 2014, IPV campaigns were implemented in Pakistan and Nigeria [4–6]. The major challenge that the Polio Eradication Initiative (PEI) program is facing now is sub-optimal coverage due to missing children. Numerous Supplementary Immunization Activities (SIAs) have been planned and implemented in the country for both OPV and IPV [4]. To stop the virus circulation, OPV campaigns have been conducted at the national and sub-national levels. IPV campaigns have been conducted only in priority areas to leverage immunity [7]. It has been demonstrated in earlier studies conducted that a fractional dose of IPV is also highly immunogenic [8, 9]. The operational feasibility of conducting a campaign with a fractional dose of IPV was already demonstrated in earlier studies conducted globally and in Pakistan [10–12]. However, community responses to different modes of IPV administration on a large scale were not reported earlier. Therefore, a special IPV campaign was conducted in Karachi in February 2019 where needle-free injectors “PharmaJet Tropis ID” were introduced for the administration of the fractional dose of IPV at a large scale. The needle-free jet injectors “PharmaJet Tropis ID” deliver vaccines intradermally by the means of a narrow, high-velocity fluid jet, which penetrates the skin and delivers the vaccine into the dermis [13]. These needle-free jet injectors have more acceptability among vaccinators due to their ease of administration, therefore, can facilitate the large-scale implementation of fIPV [10, 14]. This study aimed to analyze the impact of the needle-free device “PharmaJet Tropis ID” on vaccination status in fIPV and full IPV areas of Karachi. Furthermore, we also evaluated the effect of social mobilization activities on the awareness level of the community regarding the IPV campaign.

## Methods

### Study settings and population

Karachi is the biggest cosmopolitan city of Pakistan, which is administratively divided into six districts and 18 towns. The IPV campaign was conducted in 10 towns of five districts of Karachi from 18th – 26th February 2019 for 8 working days. In four towns including Landhi, Korangi, Liaquatabad, and North Nazimabad, a fractional dose of IPV was given using needle-free injectors “Tropis ID” by PharmaJet Company. This device is designed for intradermal injection of fractional dose up to 0.1 mL of IPV. Whereas in six towns comprising of Baldia, Bin Qasim, Gadap, Gulshan-e-Iqbal, Orangi, and SITE towns of Karachi, full-dose IPV was given to the children of 4–59 months of age. Besides, bivalent OPV (bOPV) was also administered to all children under 5 years of age in those areas.

### Data source and collection process

The total target children for fIPV were 490,806 in 43 Union Councils (UCs) and for full IPV the target children were 992, 794 in 63 UCs. Total teams required for fIPV were 744 and for full IPV 1481. The composition of each team was one skilled person, one team assistant, one social mobilizer, and one focal person (Medical Doctor) for Adverse Events Following Immunization (AEFI) at the UC level. All the team members received one full-day training. Details of vaccinator training are reported in the earlier short report [14]. The Emergency Operations Center (EOC) of Sindh province established by the Government of Pakistan was responsible for the overall coordination of the IPV campaign in Karachi with the strong support from PEI partners. To check campaign quality, independent monitors conducted at least one Rapid Convenience Assessment (RCA) house to house cluster surveys daily in areas where vaccination was already completed. They began with the first house to record vaccination status for IPV of all target-age (4–59 months) children in 10 consecutive households. In the case a household had more than one eligible child, then the data were collected from all children in respective households. The monitors collected information about the number of vaccinated and unvaccinated children in the households and reasons for unvaccinated children and the source of campaign information from parents of the children. The information was collected on the area of residence, union council, district, number of children under 5 years of age in the household, number of vaccinated children, number and reasons of unvaccinated children (parents didn’t know about the campaign, parents didn’t give importance to vaccination, the child not vaccinated due to sickness, fear of injection, fear of adverse events, distance to vaccination center, very long queue or waiting time, the child not available at home during the campaign or at the time of team visit, any other reasons). The RCA form also contained a section on the source of campaign information for the parents such as television, radio, poster, mobile miking in the area, mosque announcements in the area, community health worker’s visit, social mobilizer’s visit, and school.

After the campaign ended, RCA forms stored at district polio control rooms (DPCRs). This study used secondary data of RCA forms of the Inactivated Polio Vaccine (IPV) campaign which was collected by the Emergency Operations Center (EOC) for Polio Eradication and Immunization, Health department, Sindh Government, Pakistan in February 2019. Approval has been obtained from the ERB of Health Department Government of Sindh, Pakistan; before the acquisition of IPV campaign RCA data forms of EOC Sindh for this study.

### Data entry and analysis

The data was entered into the Microsoft Excel program and analyzed through Statistical Package for Social Sciences (SPSS) version 21. Statistical significance of associations between fractional and full IPV type with reasons for missed children and source of campaign information was determined with Pearson’s Chi-square test. *P*-value of < 0.05 was considered a statistically significant association.

## Results

**Fig. 1:**
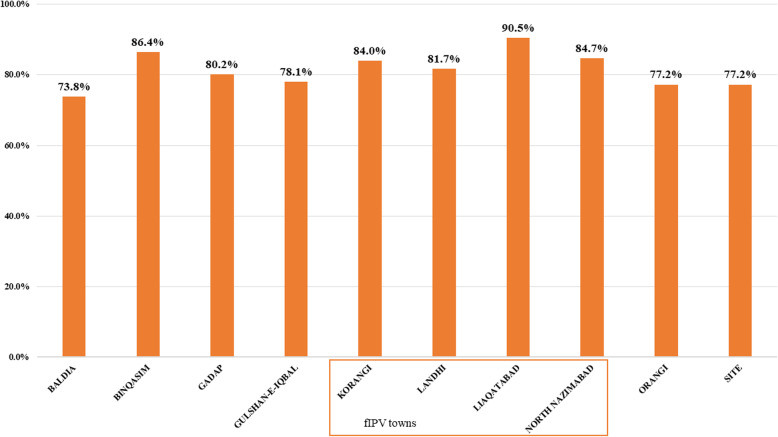
The proportion of children vaccinated in each town during the Inactivated Polio Vaccine Campaign in February 2019 in Karachi, Sindh, Pakistan

A total of 3758 RCA forms with observations of 37,580 households were retrieved from five DPCRs of the Karachi division. Among these, 3765 households were excluded due to incomplete information. Only observations from 33,815 households with IPV target children were included in the final analysis. The highest vaccination coverage was 90.5% reported from Liaquatabad town, followed by 86.4% in Bin Qasim town and 84.7% in North Nazimabad town. Whereas, the lowest vaccinated coverage 73.8% was reported from Baldia town (Fig. 1). Total of 27,650 (81.8%) children were vaccinated and 6165 (18.2%) were not vaccinated. A fractional dose of IPV was received by children of 13,274 (39.3%) households and a full dose by 20,541 (60.7%). Vaccination status by fractional and full IPV areas shows that in fIPV areas 11,327 (85.3%) children were vaccinated as compared to 16,323 (79.5%) of full-dose IPV areas.

The baseline coverage in these four fIPV towns in the previous August 2018 full-dose IPV campaign, the average coverage was 80.3% (range 77.4 to 84.7%). In the six towns receiving full-dose IPV in both campaigns, the mean coverage was 81.1% (range 73.2 to 93.7%) in August 2018. Overall, there was an increase of 5% in average coverage between August 2018 (80.3%) and February 2019 (85.3%) SIAs in fIPV areas. The coverage in six full IPV towns declined from 81.1% in August 2018 to 79.5% in February 2019. This decline may be due to low importance given to vaccination by parents, less vaccination due to the sickness of children, or due to fear of injection. The town wise comparison of the change in coverage proportion of IPV from August 2018 to February 2019 is described in an earlier report [14].

The IPV campaign is conducted once a year to boost the immunity level of children under 5 years of age in high-risk areas of Karachi. In the last August 2018 IPV campaign, about 79.4% (481,600) children were vaccinated in these 10 towns and 20.6% (125,157) children were missed from IPV dose in August 2018 SIAs.

A comparison of reasons for unvaccinated children in fIPV and full IPV areas shows that in 43 (0.3%) households, parents did not know about the campaign in fIPV areas as compared to 18 (0.1%) in full IPV areas (*p*-value < 0.0001). In 219 (1.6%) parents did not give importance to vaccination in fIPV area as compared to 864 (4.2%) in full IPV areas (*p*-value < 0.0001). More children were not vaccinated due to sickness in 1104 households (5.4%) in full IPV areas as compared to 467 (3.5%) in fIPV areas (*p*-value < 0.0001). More children were not vaccinated due to fear of injection in 370 (1.8%) in full IPV areas as compared to 91 (0.7%) in fIPV areas (*p*-value < 0.0001). Table 1 gives details of the comparison of reasons for unvaccinated children in fIPV areas as compared to full IPV campaign areas. Other variables which were insignificant in this analysis include; parents didn’t know about the place of the campaign, vaccine not available at the time of visit, vaccinator was not available at the time of visit, parents expecting that vaccinator will visit the house, and missed vaccination due to other reasons (*p*-value > 0.05).

**Table 1:** Comparison of reasons of unvaccinated by fractional and full dose Inactivated Polio Vaccine campaign areas in Karachi, Sindh, Pakistan

Variables	Fractional IPV (13274)	Full IPV (20541)	*P* value
Parents didn’t know about the campaign			
No	13,231 (99.7%)	20,523 (99.9%)	< 0.0001
Yes	43 (0.3%)	18 (0.1%)	
Parents didn’t give importance to vaccination			
No	13,055 (98.4%)	19,677 (95.8%)	< 0.0001
Yes	219 (1.6%)	864 (4.2%)	
Child not vaccinated due to sickness			
No	12,807 (96.5%)	19,437 (94.6%)	< 0.0001
Yes	467 (3.5%)	1104 (5.4%)	
Fear of injection			
No	13,183 (99.3%)	20,171 (98.2%)	< 0.0001
Yes	91 (0.7%)	370 (1.8%)	
Fear of adverse events			
No	13,217 (99.6%)	20,445 (99.5%)	0.612
Yes	57 (0.4%)	96 (0.5%)	
Vaccination center is far away			
No	13,267 (99.9%)	20,525 (99.9%)	0.386
Yes	7 (0.1%)	16 (0.1%)	
Very long queue			
No	13,257 (99.9%)	20,510 (99.8%)	0.586
Yes	17 (0.1%)	31 (0.2%)	
Other reasons of refusal to vaccination			
No	12,751 (96.1%)	19,731 (96.1%)	0.988
Yes	523 (3.9%)	810 (3.9%)	
Child not available at home during campaign			
No	12,616 (95.0%)	18,809 (91.6%)	< 0.0001
Yes	658 (5.0%)	1732 (8.4%)	

*IPV* Inactivated Polio Vaccine

Source of campaign information in fractional and full-dose IPV areas shows that more frequent mobile miking 416 (3.1%) was observed in fIPV areas as compared to 92 (0.4%) in full IPV areas (*p*-value < 0.0001). Mosque announcements were also more rigorous 1247 (9.4%) in fIPV areas as compared to 542 (2.6%) in full IPV areas (*p*-value < 0.0001). In contrast more Community Health Workers (CHWs) 11,210 (54.6%) visited areas of full IPV campaign as compared to 4829 (36.4%) visits in fIPV areas (*p*-value < 0.0001). Whereas an additional 4122 (31.1%) social mobilizers visited fIPV areas as compared to 4675 (22.8%) in full IPV areas (*p*-value < 0.0001). Other sources of campaign information such as newspapers, neighbors, or relatives, and any other were statistically insignificant in this analysis. Table 2 shows details of a comparison of the source of campaign information in a community in fIPV areas as compared to full IPV campaign areas.

**Table 2:** Comparison of source of campaign information among fractional and full dose Inactivated Polio Vaccine campaign areas in Karachi, Sindh, Pakistan

Variables	Fractional IPV (13274)	Full IPV (20541)	*P* value
Television			
No	13,232 (99.7%)	20,435 (99.5%)	0.007
Yes	42 (0.3%)	106 (0.5%)	
Radio			
No	13,253 (99.8%)	20,519 (99.9%)	0.198
Yes	21 (0.2%)	22 (0.1%)	
Poster			
No	13,173 (99.2%)	20,509 (99.8%)	< 0.0001
Yes	101 (0.8%)	32 (0.2%)	
Mobile miking			
No	12,858 (96.9%)	20,449 (99.6%)	< 0.0001
Yes	416 (3.1%)	92 (0.4%)	
Mosque announcements			
No	12,027 (90.6%)	19,999 (97.4%)	< 0.0001
Yes	1247 (9.4%)	542 (2.6%)	
Community health worker visit			
No	8445 (63.6%)	9331 (45.4%)	< 0.0001
Yes	4829 (36.4%)	11,210 (54.6%)	
Social Mobilizer visit			
No	9152 (68.9%)	15,866 (77.2%)	< 0.0001
Yes	4122 (31.1%)	4675 (22.8%)	
Schools			
No	13,037 (98.2%)	20,345 (99.0%)	< 0.0001
Yes	237 (1.8%)	196 (1.0%)	

*IPV* Inactivated Polio Vaccine

## Discussion

This is the first study that has compared vaccination coverage and community response at the household level by full IPV and fractional IPV doses from campaign monitoring data of the polio-endemic region. Overall, 82% of children received IPV during February 2019 campaign and it ranged from a minimum of 74% to a maximum of 91% in different towns of Karachi. The previous study on a fractional dose of IPV campaign was conducted in October 2016 in four districts of Hyderabad division, Sindh province also reported 82% coverage based on parent’s recall [15]. In this study, the BCG syringe has been used for the administration of the fractional dose of IPV in Hyderabad. Whereas in fractional dose IPV areas more children 85% were vaccinated as compared to 80% of children vaccinated in IPV full dose areas of Karachi. The use of needle-free injectors for IPV administration in fractional dose areas can be one of the reasons for this difference in fIPV coverage in Hyderabad and Karachi. The use of needle-free injector Tropis ID for the administration of fIPV in a campaign setting was shown to be feasible, safe, and efficient in earlier few small studies in Pakistan [16, 17]. However, the previous study was conducted on a small scale with a sample size of 582 households in one low-income area of Bin Qasim town of Karachi [16]. In another study conducted in four low-income peri-urban areas of the same Bin Qasim town of Karachi, fIPV was administered through three different devices to 308 children [17]. High vaccination coverage of 94% during the fractional dose IPV campaign was also achieved in campaign settings in India [18]. In India, the fIPV campaign was conducted in June 2016 as an outbreak response to vaccine-derived poliovirus type 2 (VDPV2) in two districts [18]. Moreover, they have used a BCG syringe for the administration of the fractional dose of IPV, which requires the technical expertise of the vaccinator. Whereas > 95% IPV coverage was also noted in Kenya where combined IPV and OPV were administered to children aged < 59 months during the campaign in December 2013 in five divisions [2]. High coverage noted in these studies could be due to a difference in study settings and population. Feasibility and acceptability of combined OPV and IPV were already established through randomized controlled trials conducted in the Gambia, Oman, Thailand, Cuba, Sri Lanka, and Bangladesh [10–12, 19, 20].

Our results showed that a significant number of parents did not know about the IPV campaign in fractional dose IPV areas as compared to full dose IPV areas. Whereas significantly more parents did not give importance to vaccination, children were not vaccinated due to sickness, due to fear of injection, and due to unavailability of children at home during campaign days in full dose IPV areas as compared to fractional dose IPV areas. Pervaiz et al. also reported refusals, fear of injections, lack of awareness, and absence of child at home during the campaign days as the main reasons for children not being vaccinated in the October 2016 fractional IPV campaign in Hyderabad division [15]. Among the unvaccinated children recorded in Kenya during the IPV campaign, about 46% of caregivers were not knowing about the place of vaccination, 15% missed due to refusal, and 9% of children were not available during the IPV campaign [2]. Whereas major reasons for no vaccination included child was not available on the day of vaccination 29%, followed by the child was sick 21% and lack of parental awareness 16% were reported in India. Furthermore, hesitancy and refusal of 6% and fear of injection 2% were also noted in the Indian study, also observed in our study [18]. The vaccine hesitancy is also influenced by fear of injection, fear of side effects of the vaccine, and educational attainment of the parents as observed in a cross-sectional study conducted in Indonesia [21].

Social mobilization is one of the key strategies to enhance community awareness regarding the IPV campaign [22]. We have also reported the effect of communication and social mobilization strategies on community acceptance of the injectable vaccine campaign in Karachi. Overall better social mobilization (mobile miking, Mosque announcements, and visits by social mobilizers) in fractional dose IPV areas as compared to full-dose IPV areas was noted in this campaign in Karachi whereas it was reported as suboptimal in 2016 during the IPV campaign in Hyderabad [15]. Strong social mobilization measures were effective in achieving high coverage in other settings like India and Kenya. Like our study, the most common source of campaign information among caregivers in Kenya were megaphone announcements 76% followed by a visit of a social mobilizer 47%, visits of health care workers 43, and 36% radio announcements [2, 18].

Our study has certain limitations and strengths. It is an observational study and it is also possible that some parents might not have provided actual reasons for refusing to vaccinate their child. The main strengths of our study include a large sample size which can determine statistical significance between small proportions as well. Moreover, due to the random selection of households as per standard World Health Organization (WHO) RCA survey methodology, the occurrence of selection bias is less likely. Besides, this is the first study that showed a comparison of community response towards two different modes of IPV vaccine administration. The study findings support the use of intradermal needle-free injectors because of some factors such as the low quantity of vaccine required, easy to administer by the vaccinators, and willingness of parents due to painless administration of the vaccine. These factors may contribute to the low cost of this method, but we have not focused on cost analysis in this study. However, another future study can highlight the cost-effectiveness of this method by including the attributable proportion of increase due to fIPV, mobile miking, and fear of injection.

## Conclusions

It is concluded that a fractional dose of IPV by using needle-free injector “Tropis ID” for the administration of fIPV in a campaign setting is well accepted by the community. We have reported the source of campaign information in the community and reasons related to a misconception that can be dealt with for planning a successful campaign in the future. Besides, vigorous social mobilization through mobile miking, Mosque announcements, social mobilizer’s visits, and CHW visits help achieve high IPV vaccination coverage. Our analysis supports the use of IPV in mass campaigns to maintain an immunity level that would diminish the number of paralytic cases in the occurrence of endemic transmission of poliovirus as good vaccination coverage is feasible especially by using a fractional dose of IPV.

## Data Availability

The datasets used and/or analyzed during the current study are available at the EOC Sindh (sindh.eoc@gmail.com) on a reasonable request.

## Abbreviations

IPV: Inactivated Polio Vaccine
fIPV: The fractional dose of Inactivated Polio Vaccine
OPV: Oral Polio Vaccine
PEI: Polio Eradication Initiative
SIAs: Supplementary Immunization Activities
bOPV: Bivalent Oral Polio Vaccine
UC: Union Councils
AEFI: Adverse Events Following Immunization
EOC: Emergency Operations Centre
RCA: Rapid Convenience Assessment
DPCRs: District Polio Control Rooms
SPSS: Statistical Package for Social Sciences
VDPV2: Vaccine-derived Poliovirus Type 2
WHO: World Health Organization
CHWs: Community Health Workers
